# LYMPHOCYTE IMMUNOSUPPRESSION AND DYSFUNCTION CONTRIBUTING TO PERSISTENT INFLAMMATION, IMMUNOSUPPRESSION, AND CATABOLISM SYNDROME (PICS)

**DOI:** 10.1097/SHK.0000000000001675

**Published:** 2021-06-01

**Authors:** Christian B. Bergmann, Nadine Beckmann, Christen E. Salyer, Peter A. Crisologo, Vanessa Nomellini, Charles C. Caldwell

**Affiliations:** * Division of Research, Department of Surgery, College of Medicine, University of Cincinnati, Cincinnati, Ohio; † Division of Podiatric Medicine and Surgery, Critical Care, and Acute Care Surgery, Department of Surgery, College of Medicine, University of Cincinnati, Cincinnati, Ohio; ‡ Division of Trauma, Critical Care, and Acute Care Surgery, Department of Surgery, University of Cincinnati, Cincinnati, Ohio; § Division of Research, Shriners Hospital for Children, Cincinnati, Ohio

**Keywords:** B cells, double-negative T cells, immunosuppression, innate lymphoid cells, natural killer T cells, regulatory T cells

## Abstract

Persistent Inflammation, Immune Suppression, and Catabolism Syndrome (PICS) is a disease state affecting patients who have a prolonged recovery after the acute phase of a large inflammatory insult. Trauma and sepsis are two pathologies after which such an insult evolves. In this review, we will focus on the key clinical determinants of PICS: Immunosuppression and cellular dysfunction. Currently, relevant immunosuppressive functions have been attributed to both innate and adaptive immune cells. However, there are significant gaps in our knowledge, as for trauma and sepsis the immunosuppressive functions of these cells have mostly been described in acute phase of inflammation so far, and their clinical relevance for the development of prolonged immunosuppression is mostly unknown. It is suggested that the initial immune imbalance determines the development of PCIS. Additionally, it remains unclear what distinguishes the onset of immune dysfunction in trauma and sepsis and how this drives immunosuppression in these cells. In this review, we will discuss how regulatory T cells (Tregs), innate lymphoid cells, natural killer T cells (NKT cells), TCR-a CD4^−^ CD8^−^ double-negative T cells (DN T cells), and B cells can contribute to the development of post-traumatic and septic immunosuppression. Altogether, we seek to fill a gap in the understanding of the contribution of lymphocyte immunosuppression and dysfunction to the development of chronic immune disbalance. Further, we will provide an overview of promising diagnostic and therapeutic interventions, whose potential to overcome the detrimental immunosuppression after trauma and sepsis is currently being tested.

## DEFINITION OF PICS

Many septic and severe trauma patients require treatment in the intensive care unit (ICU). Due to improved clinical care in recent years, however, acute mortality rates have decreased in these patients (1). Nevertheless, death rates are still unacceptably high and current research shows that even survivors often have prolonged ICU stays, struggle with recovery after discharge, and fail to regain their strength (2). For the development of better diagnostics and therapies for this condition, it became necessary to define it more clearly. In 2012, Gentile et al. (2) coined the term PICS: Persistent Inflammation, Immunosuppression and Catabolism Syndrome and defined the clinical determinants: prolonged hospitalization (> 14 days), inflammation (C-reactive protein levels > 150 μg/dL), immune suppression (lymphocyte count < 800/mm^3^), and weight loss despite adequate nutrition (loss of lean body mass > 10% during hospitalization or BMI < 18 and, serum albumin < 3.0 g/dL) (2, 3). The profound impact of the disease, however, becomes evident when looking at the time after hospitalization: many PICS patients require treatment in long-term acute care facilities—Rosenthal and Moore (4) report that by 1 year after ICU discharge, 50% of PICS patients have died and another 25% remain bedridden. This inability to properly recover is especially observed in older patients, who suffer from persistent inflammation more frequently than younger adults (5). With medical and technical progress making it more likely to survive the initial inflammatory insult, the aging of Western societies thus makes it evident that there is a dire need for a better understanding of the prolonged immune-imbalance underlying PICS (2). The focus of this review is set on the contribution of lymphocytes to the development of PICS after trauma and sepsis; however, PICS can also be found in ICU patients suffering from other diseases.

## IMMUNOSUPPRESSION AND IMMUNE CELL DYSFUNCTION IN PICS

PICS is characterized by concurrent inflammation and immunosuppression. Currently, it is not fully understood why these apparently contradictory conditions co-exist and how this is regulated.

Danger-associated molecular patterns (DAMPs) and pathogen-associated molecular patterns (PAMPs) can drive inflammation in response to sterile or non-sterile tissue damage (6, 7). Recognition of DAMPs and PAMPs via Toll like receptors (TLRs) causes activation of the innate immune system, especially neutrophils and macrophages (8, 9). These, among other cells, maintain inflammation by producing pro-inflammatory cytokines, i.e., tumor necrosis factor alpha (TNF-α), interleukin 6 (IL-6), IL-8, monocyte chemoattractant protein 1 (MCP-1), and macrophage inflammatory protein 1 alpha (MIP1α) (10, 11). In particular, IL-6 plays a significant role as it potently promotes the production of acute phase proteins, immune cell maturation and activation, and directly correlates with the severity of injury (12, 13).

Compared to the well-characterized effects of these proinflammatory cytokines, the immunosuppressive effects of anti-inflammatory cytokines are poorly understood. Prominent anti-inflammatory cytokines in trauma and sepsis are interleukin-10 (IL-10) and transforming growth factor beta (TGF-β) (14-16). Newer studies also attribute an important role to thymic stromal lymphopoietin (17, 18). All three cytokines significantly change immunologic functions in innate and adaptive immune cells. In the context of trauma and infection, these changes contribute to an immunosuppressive state that is marked by T cell exhaustion (19, 20), increased proportions of regulatory T cells (Tregs) directly after trauma or onset of sepsis (21, 22), paralysis of monocytes, macrophages, and dendritic cells (2, 23, 24), neutrophil dysfunction (25), and stark expansion and activation of immunosuppressive myeloid-derived suppressor cells (MDSCs) (26).

## CONTRIBUTION OF LYMPHOCYTES TO THE DEVELOPMENT OF PICS

Adaptive immune cells are potent regulators of myeloid innate immune cell functions in sepsis (27-30). An important component of PICS, which is also the part of the clinical definition (lymphocyte count < 800/mm^3^), is lymphopenia (2). It is prevalent at admission and can remain for up to 30 days (31). In a murine PICS model we could previously show that lymphopenia is not only depicted in a quantitative loss of T cells but, but also qualitative impairment occurs as naive CD4 and CD8 T cell numbers are also dramatically decreased (20). This is of significance as studies did reveal that persistent lymphopenia is related to increased mortality and secondary infections in severely ill intensive care patients (32-34). Moreover, first promising approaches to treat septic patients with IL-7, a cytokine modulating the survival and proliferation of T cells (NCT02640807), underline the importance of lymphocytes in the development of chronic immune impairment in PICS. Current literature focusses to a great extent on the role of MDSCs in the development of PICS (3, 35).

In this review, we will focus on immunosuppression through Tregs, as they are the most potent immunosuppressive member of the T-cell family (28), show a relative increase in the T-cell population in sepsis and hempen, e.g., CD4 T-cell function (27, 28). The impairment of effector T-cell function in sepsis was nicely elsewhere (36, 37). In addition, several studies suggest a key role of Tregs in chronic immunosuppression in critically ill patients (38-40). Moreover, we sought to fill a gap in the understanding of the contribution of lymphocyte immunosuppression and dysfunction to the development of chronic immune disbalance. A growing body of evidence suggests that a number of different lymphoid cell populations have significant immunosuppressive functions, but are less commonly reviewed: innate lymphoid cells (ILC), natural killer T cells (NKT cells), TCRαβ^+^ CD4^−^ CD8^−^ double-negative T cells (DN T cells), and B cells (41). As a conclusion their contribution to chronic immune disbalance in PICS might be underestimated or at least is not yet well characterized. With this review we provide an overview about the most important immunosuppressive functions and dysfunctions that might lead to the development of PICS.

## PROGRESSION FROM SEPTIC OR TRAUMATIC INSULT TO PICS

We suggest that in the case of trauma and sepsis the initial insult already initiates the development of PICS. Clinical indications for this suggestion are that in sepsis key immunosuppressive mechanisms were shown to be upregulated already at the onset of sepsis (within 24 h: upregulation of IL-10 serum levels (35), lymphopenia and T-cell dysfunction (in septic shock) (42), increase of serum levels of MDSCs (3); within 3–5 days: relative increase and activation of Tregs (38, 43)) and remain throughout the development of chronic critical illness (3, 34, 35, 38). Moreover, some studies displayed a correlation between initial quantitative and qualitative cell dysfunction and the development MODS (44, 45), which can be part of PICS. Therefore, we reviewed acute and chronic changes of lymphocyte behavior, which we believe will help evaluate development of PICS in early stages.

Patients developing chronic critical illness such as PICS are a severe burden for the healthcare system due to a high mortality, especially in septic patients (cumulative mortality after 2 years between 67% (46) and 42% (47)). Interestingly not all patients develop chronic immune disturbances such as PICS. A recent publication of Hawkins et al. (35) showed that patients in a septic cohort can either progress to early death (<14 d), rapid recovery (6-month survival of 98%), or chronic critical illness (6 month survival of 63%). Currently, there is no diagnostic tool to evaluate in which of these categories a patient will progress. Different clinical phenotypes of sepsis have been described, as an attempt to distinguish different immune disturbance patterns, affecting different organs (48). This might guide treatment and allow estimating the chances of survival. We believe that beside the clinical evaluation defining the clinical phenotype, the assessment of cellular phenotypes mirroring the immunologic state of lymphocyte cell is important to guide diagnostic and treatment. Cellular phenotypes can be examined using different biomarkers such as surface receptors, cytokines, or combinations thereof. Several studies displayed better predictive values when biomarkers were combined (49, 50). Functional assays of biological activity seem promising in the attempt to gather a more valid picture of immunosuppressive functionality (51), which might not be fully reflected by the measurement of biomarkers.

As a consequence, it might be possible to adjust therapeutic approaches that failed in the past because treatment was not adjusted to the patients’ immune status. Potential diagnostic and therapeutic approaches are therefore highlighted in the last chapters.

## LYMPHOCYTE IMMUNOSUPPRESSION AND DYSFUNCTION IN TRAUMA, SEPSIS ITS CONTRIBUTION TO PICS

### Regulatory T cells (Tregs)

#### Types of tregs—

The origin of Tregs allows a broad categorization as either thymus-derived (tTreg) or peripherally derived (pTreg). Immune tolerance to self-antigens presented in the thymus is provided by tTregs, whereas pTregs ensure peripheral immune tolerance to non-pathologic foreign antigens, such as commensal bacteria in the gastro-intestinal tract (52) or antigens experienced during pregnancy in the placenta (53). These antigens cannot be presented in the thymus. Thus, peripheral immune tolerance has to be ensured by pTregs, in addition to serving to regulate innate and adaptive immune cells in inflammation (54, 55).

One well-studied sub-group of pTregs are Type 1 regulatory T cells (Tr1 T cells) (54, 55). Induction of Tr1 T cells occurs in the periphery. Currently there is no specific transcription factor that controls differentiation or characterizes Tr1 T cells (54). They do not constitutively express FoxP3, which distinguishes Tr1 T cells from FoxP3-expressing pTregs (54). The functional differences between FoxP3^+^ pTregs and FoxP3^−^ Tr1 T cells have not yet been systematically examined. Roncarolo et al. (56) observed different unsupervised transcriptomics and found that murine Tr1 T cells seem to rely on aerobic glycolysis whereas FoxP3^+^ Tregs metabolism is dependent on oxidative phosphorylation. Both cell types are found in secondary lymphoid tissue and there seems to be a functional interaction between Tr1 T cells and FoxP3^+^ Tregs (56). A distinct cytokine profile of TGF-β and high levels of IL-10 expression can characterize Tr1 T cells (57, 58), as well as the expression of the surface markers CD49b and LAG-3 (59). However, the specificity of CD49b and LAG-3 to identify Tr1 T cells has been questioned by some authors, as some other cell types, including CD8 T cells and B cells, were shown to express them as well (60, 61).

The majority of Tregs in the lymphatic system are believed to be tTregs (~70–90%) (62, 63). However, assessing this distribution is difficult, as it is still unclear how to adequately distinguish tTregs and pTregs experimentally. Shevach and colleagues identified the transcription factor Helios and the cell surface molecule neuropilin-1 (NRP1), as distinguishing markers (64). However, the specificity of these markers has also been questioned and thus their use remains controversial (65).

In addition to their origin, Tregs can be categorized further by their similarities in differentiation to non-regulatory T-cell lineages (Table 1). These distinctions are important, as it is becoming clear that these cell types fulfill different regulatory functions and affect different T cells. Table 2 summarizes the different immunosuppressive capabilities of Tregs with respect to their life cycle, into “central” cTregs, “effector” eTregs and “memory” mTregs. These cells seem to have different functions in different tissues, almost like a division of labor (66): cTregs circulate throughout secondary lymphatic tissues, dependent on IL-2 to maintain homeostasis and suppress T-cell priming (66). On the other hand, eTregs mainly reside in non-lymphatic tissue, their homeostasis and proliferation are largely IL-2 independent and they provide rapid immunosuppression in inflamed tissue (66, 67). The last subtype, mTregs, also resides in non-lymphatic tissue but maintains regulatory function at barrier surfaces, such as the skin. While mTregs develop after transient Ag recognition, eTregs need TCR-mediated signaling for self-maintenance (66).

Another distinction of Tregs, that is gaining importance, is their localization. A growing body of evidence shows that Tregs serve to maintain homeostasis in different tissues (68). A recent study shows that human Tregs from blood, lung, and colon show distinct receptor expression patterns at the mRNA level (69). While these patterns still have to be confirmed on protein level, they indicate that Tregs from different tissues may be functionally distinct. Another study supports this notion as they characterized Treg subsets by their characteristic transcription factor expression and cytokine release and found distinct distribution patterns in different organs (70).

In line with this, another interesting challenge is enumeration of the concentration of Tregs in different compartments. If tissue-resident and circulating Tregs are indeed functionally distinct, they may have unique contributions to the immune response to sepsis and trauma. Importantly, if a higher percentage of Tregs than previously thought is tissue-resident, then these cells may actually produce the majority of immunosuppressive cytokines released during trauma and sepsis, and not their more frequently studied, circulating counterparts. In any case, how Treg functions and plasticity are affected by trauma and sepsis is not yet fully understood. Moreover, as trauma can be considered a sterile inflammation (at least in its early stages), the mechanisms of Treg activation in trauma and sepsis are likely to be different. But the discussed- and future categorizations of Tregs will help elucidate their role in systemic inflammation in both trauma and sepsis.

#### Immunosuppressive functions of Tregs—

Tregs exert immunosuppressive functions on other T cells, dendritic cells, B cells, macrophages, osteoblasts, mast cells, NK cells, and NKT cells by a variety of methods (71). For instance, Tregs can produce IL-10, IL-35, and TGF-β, which suppress CD4^+^ effector T cells and antigen-presenting cells (APCs) (27, 28, 54, 72). Further, adjacent effector T cells can be starved by Tregs by upregulating their own IL-2 receptor (CD25) expression and thus scavenging IL-2 (27, 28). Moreover, Tregs can directly destroy effector T cells and B cells via cytolysis with granzymes (27, 28). Myeloid APCs can be destroyed via the release of granzyme B and perforins and via cell–cell contacts through CD2/CD58 and CD266/CD155 (54, 73, 74). In terms of naive T cells, Tregs can hamper their activation by impeding their interaction with dendritic cells (27, 75). Metabolically, Tregs can affect T cells by hydrolyzing extracellular ATP to adenosine with CD39 and CD73 (54, 76). Adenosine is known to suppress T-cell activation and function through adenosine A2a receptor (77). For an in-depth review of Treg immunosuppressive functions, please refer to Caridade, Graca, and Ribeiro (27).

#### Treg-mediated immunosuppression in trauma and sepsis—

The role of Tregs in trauma and sepsis is still only partially understood. Generally, Tregs are thought to help restore immune-homeostasis through their immunosuppressive functions, and thus Tregs are typically considered protective in the context of trauma and sepsis (78, 79). However, it is not yet known how clinically relevant changes in Treg quantities and functions are with regard to patient outcomes and if these can be used to predict the clinical course. Particularly in patients with a prolonged disease course, such as PICS, immunosuppression through Tregs could conceivably contribute to poor outcomes (79). Some studies have started to investigate this issue.

#### Preclinical studies—

Preclinical animal studies suggest that higher Treg numbers are associated with increased mortality: recently, Hu et al. (80) showed reduced disease severity and increased survival in a murine two-hit sepsis plus pneumonia model upon *in vivo* Treg depletion with anti-CD25 antibody. Increased survival was also reported in a murine sepsis model following indirect reduction of Tregs through IL-10 and TGF-β neutralization (81). Other Murine studies also found that the frequency of Tregs increases in the acute phase of sepsis, while the total number of CD4^+^ T cells concomitantly decreases (82). It is still unclear if this increase in Treg frequency is solely due to the apoptosis of conventional T cells or if an expansion of Tregs is induced as well.

#### Clinical studies—

Similar dynamics have been observed in human patients. In a prospective observational study including 106 burn patients, some of which developed sepsis, Treg frequency in peripheral blood was positively associated with the size of the burn area, as were IL-10 and TGF-β levels (38). Additionally, FoxP3 and CTLA4 expression levels were increased in Tregs of non-survivors with sepsis (38). In another study of patients with post-traumatic sepsis, an imbalanced Th17/Treg ratio positively correlated with an increased sequential organ failure assessment (SOFA) score (83). Among septic ICU patients, non-survivors had a significantly increased Treg ratio by day 7 (39). Additionally, the INFECT trial suggests that an increased Treg ratio predicts susceptibility to secondary infections in ICU patients (40). These findings contrast the general assumption that Tregs are beneficial in trauma and sepsis but rather suggest, as seen in preclinical studies, that increased Treg numbers are associated with worse outcomes.

#### Consequences for the role of Tregs in PICS—

These findings highlight the gaps in our understanding regarding Tregs in trauma and sepsis, but much less in PICS. Further studies are needed to determine how Treg proportions and functions change in response to these conditions. Future studies will address if the increases in Treg ratios observed so far are the result of a reactive release, i.e., the host’s attempt to control an overwhelming excessive inflammation, or rather a proportional shift resulting from the death of conventional T cell and/or dysfunctional proliferation of Tregs. The latter may be promoted by the high basal proliferation rate of Tregs compared to conventional T cells (84). Additionally, the contribution of different types of Tregs and their origin will have to be considered. Many clinical studies with Tregs from peripheral blood use CD4^+^ and CD25^+^ as markers and lack differentiation in “central” cTregs and “effector” eTregs, which seem to have different functions in different tissues (66). Answers to these questions will help develop strategies to stratify patients and may subsequently allow for therapeutic targeting of Tregs in a personalized- and Treg-subset specific manner, which has great potential for improving patient outcomes.

### Innate lymphoid cells (ILCs)

#### Types of ILCs—

Innate lymphoid cells (ILCs) are derived from common lymphoid progenitors, but unlike T or B cells, they lack an antigen-specific T- or B cell receptor. Originally, they were grouped as equivalents to T-cell lineages as ILC1 (Th1), ILC2 (Th2), and ILC3 (Th17). Since then, two more classes of ILCs have been described, NK and lymphoid tissue inducer (LTi) cells (85). The nomenclature and function of ILCs is summarized in Table 3. ILCs regulate immune homeostasis in different tissues. ILC3s, for example, maintain control over symbiotic microbiota (85). In contrast to T cells, ILCs respond immediately to inflammation, much like innate immune cells. Similar to their “mature” T-cell relatives, however, ILCs also show great plasticity, enabling them to change their phenotype if stimulated by certain cytokines (85).

Unlike lymphocytes, ILCs are considered local keepers of tissue function: most ILCs reside in tissues long-term and do not circulate through the blood. Locally ILCs are maintained by self-renewal and can expand upon systemic perturbations of immune homeostasis (86). The exceptions are NK cells and inflammatory ILC2s, which are found in relevant numbers in peripheral blood. NK cells make up 5% to 15% of lymphocytes in human blood. They are potent producers of interferon gamma (IFN-γ) and TNF-α and augment functions of macrophages and dendritic cells. Additionally, NK cells can kill target cells through death ligand engagement and release of perforins and granzymes (29).

#### NK cells in trauma and sepsis—

Very few studies have examined the role of ILCs in sepsis so far. Thus, the underlying mechanisms leading from normal hemostatic functions of ILCs to their deleterious dysfunction in trauma and sepsis are not well understood. In case of NK cells, it is well established that they express several TLRs (2, 4, 7, and 9 (87)). Their degree of activation could thus result from the specific bacterial strain and the tissue from where the infection originates.

#### Preclinical studies—

To date, most research on the matter has focused on decreased NK cell numbers and their impaired IFN-γ production. Some studies report a suppression of NK cells during sepsis. Reduced cytotoxic activity and cytokine production by NK cells, as well as increased expression of programmed death receptor 1 (PD1) and its ligand PD-L1, have been described in septic mice (88).

An important role of IFN-γ production by NK cells was indicated by a sepsis study in rats that showed increased NK cell numbers, serum IFN-γ levels, and survival after IL-15 treatment (89). This study and others suggest that NK cells are significant producers of IFN-γ in sepsis (89, 90). In contrast to the previously described results on suppression of NK cells during sepsis, NK cell numbers were reported to increase in mesenteric lymph nodes, colon, and ileum on day 7 in a murine sepsis model (91) which roughly coincides with the day that mice develop PICS (20). Further, the depletion of NK cells in a rodent sepsis model resulted in decreased mortality (29). Although IFN-γ typically amplifies microbial clearance, the improved survival in this study was attributed to mitigation of an excessive release of IFN-γ (29). Results from a murine endotoxemia study indicate that IFN-γ production by NK cells, which was induced through mTORC1 by activated invariant NKT cells, reduces macrophage phagocytic capacity, impairs clearance of secondary Candida infection, and increases mortality (30).

#### Clinical studies—

The suggestion that murine NK cells with increased PD-1 expression supposedly play a detrimental immunosuppressive role (88) was confirmed in humans. A correlation between increased PD-1 expression on NK cells in septic patients and reduced monocyte and neutrophil phagocytic functionality has been described (92).

**Fig. 1.**
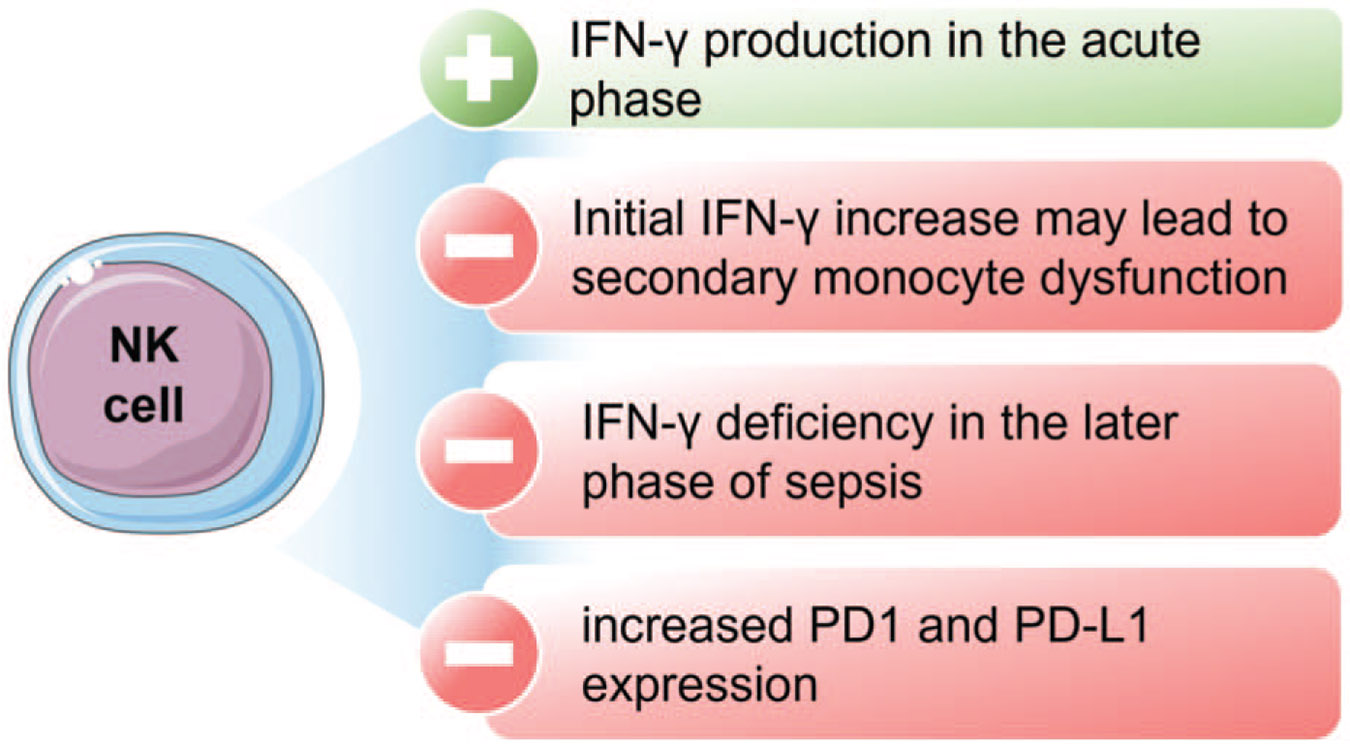
Immunosuppressive functions of natural killer cells (NK cells). Natural killer cells promote the acute immune response through production of IFN-γ. The initial increase of IFN-γ however may lead to secondary monocyte dysfunction. In the course of sepsis a decrease of IFN-γ can be seen as well as an increase in PD-1 and PD-L1 expression, impairing proper immune responses by NK cells.

In humans, similar to mice, impaired IFN-γ production by NK cells is thought to contribute to the deleterious effects of immunosuppression after burn injury (93). The effects on the inflammatory response are visualized in Figure 1. In line with this, several clinical studies report beneficial effects of supportive IFN-γ treatment in sepsis (94, 95) and trauma (96). Individual clinical cases also report improved outcomes in severe infection (97), but larger clinical studies are still needed. Impaired NK cell IFN-γ was also associated with cytomegalovirus reactivation in critically ill patients (98). In fungal infection the dynamics of IFN-γ release seems to be important. In a case series, using IFN-γ as treatment for invasive fungal infections some immune functions could be restored (99). However, high IFN-γ levels in the early state of sepsis in human patients, were reported to be associated with the development of secondary Candida infections (30). These findings are important to examine in future studies as secondary infections are a clinical expression of chronic immunosuppression that can be seen in PICS (2).

It is well established in both human and mice that TGF-β can downregulate IFN-γ release and impair cytolytic functions of NK cells (15). Thus, these findings may also contribute to our understanding of how TGF-β release contributes to immunosuppression in trauma and sepsis. This is a point of contention, however, as some researchers argue for a role of TGF-β and IL-10 in modulating NK cell responses in sepsis and trauma (100), while others report no- or only minimal effects (93).

Consequences for the role of NK cells in PICS—These contrasting results indicate that NK cell functions are neither detrimental nor beneficial in sepsis and trauma *per se*, but depend on the complex dynamics of the host’s response in the respective tissue. Future research might focus more on tissue-specific inflammatory responses of NK cells and on the role of distinct NK subsets. In humans, for instance, it is possible to distinguish a CD56^dim^ CD16^bright^ cytotoxic subset and a CD56^bright^ CD16^−/dim^ subset, which predominantly produces cytokines (29). We therefore suggest that if a rather suppressive or dysfunctional NK cell subtype relatively dominates over time, or the acute or hyperacute activation of NK cells impairs other cells permanently, NK cells might therewith contribute to the development of PICS. Differences in NK cell behavior between mice and humans also hinder our understanding of their role in sepsis and will have to be addressed in future studies as well (29).

#### Other ILCs in trauma and sepsis—

Our knowledge on the role of other ILCs is still limited. Similar to NK cells, findings regarding their beneficial or detrimental role in sepsis and trauma vary substantially. Exact activation mechanisms still have to be defined for other ILCs other than NK cells, but a variety of cytokines are known to modulate ILC function (29, 100, 101) and a dependency on micronutrients and Vitamin A have been described (85). Thus, an impaired metabolic and nutritional state, as present in PICS, may contribute to ILC dysfunction.

#### Preclinical studies—

ILCs are generally considered to play an important role in tissue repair, especially ILC3s, which contribute to immune hemostasis in the gut (102). An increase in ILC2 cells has been described as harmful in allergic airway inflammation in mice as they promote Th2-associated cytokines (103). Similarly, a harmful role was described in the gut. In *Enterococcus faecalis* translocation after burn injury, murine ILC2 cells in the lamina propria of the intestine were reported to impair antibacterial defenses (104). On the other hand, ILC2 cells protected lung endothelial cells from pyroptosis in a murine sepsis model (105). The same study also reported an increase of ILC2 cells after sepsis induction (105). A tissue-dependent upregulation of ILC2 cells was also observed in mice and humans with abdominal sepsis, but in this study ILC2 depletion in a murine cecal ligation and puncture sepsis model was associated with a beneficial outcome (106).

Although increased proliferation was also reported for lung ILC2 s, as well as for NK, ILC2, and ILC3 cells, in a murine two-hit model of sepsis plus *Pseudomonas pneumonia*, IL-7 treatment led to improved survival (107). In this study, cytokine levels such as IFN-γ were unchanged in blood and spleen, but elevated in the lung. It was suggested that during the early onset of sepsis, the proliferation of ILCs might be of higher importance than the proliferation of other lymphoid cells, such as CD4 and CD8 T cells, as the latter need more time to orchestrate pathogen defenses (107).

#### Clinical studies—

**Fig. 2.**
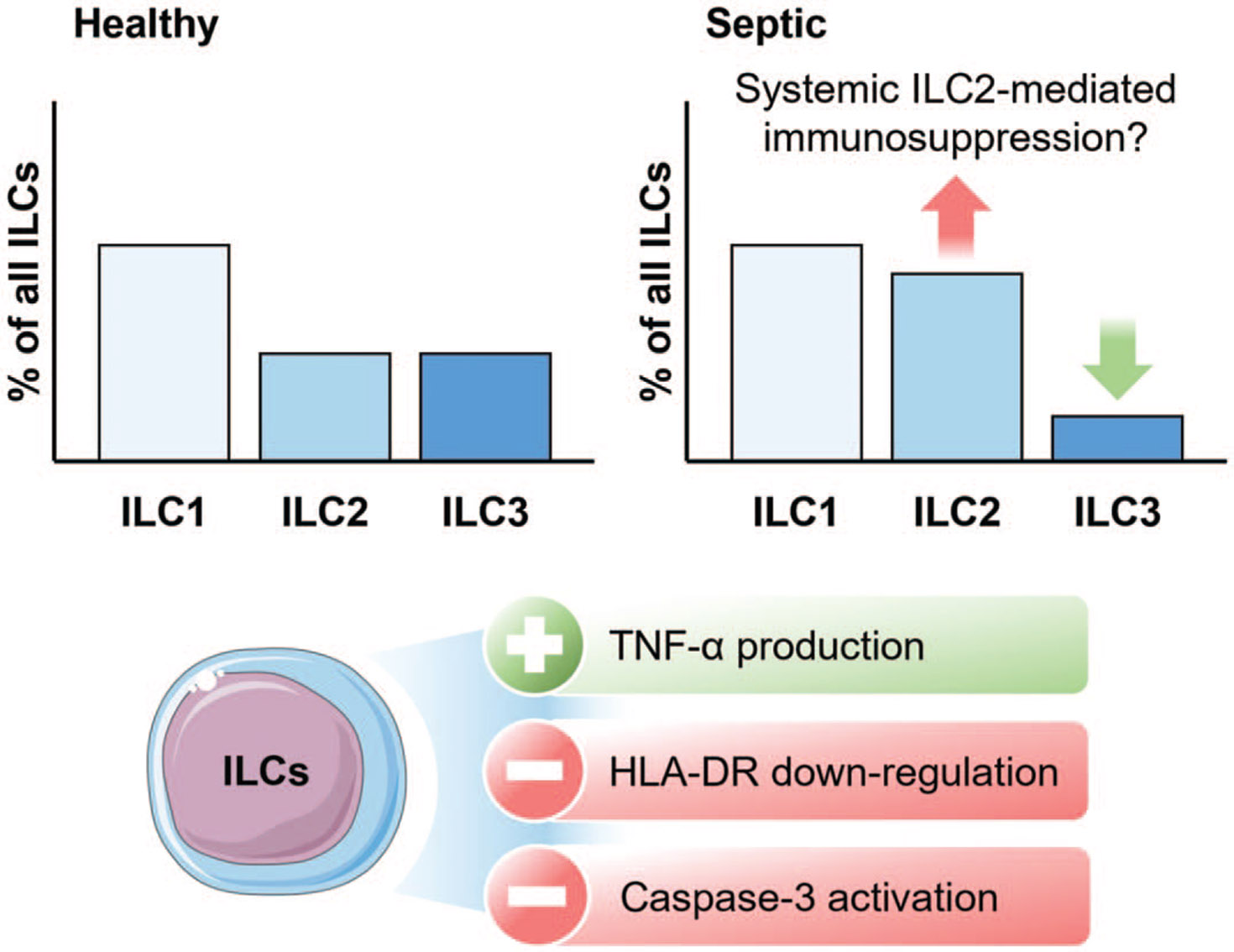
Functions and relative shift in proportion of innate lymphoid cells (ILCs). In sepsis a relative shift of the proportions of innate lymphoid cells can be observed. This suggests that ILC2 contribute to the immunosuppressive state post-sepsis. Moreover, HLA-DR expression and Caspase-3 activation indicate decreased function and increased susceptibility to apoptosis impairing the immune response by ILCs. However, the ability to produce pro-inflammatory TNF-α seems mostly unaffected.

A proportional increase of ILC2 cells compared with ILC3 cells was reported in the peripheral blood of septic patients (108). In this case, however, the increase appeared to result from apoptosis of circulating ILC1 and ILC3 cells. All ILCs showed increased expression of active caspase 3, an apoptosis marker, leading to an overall decrease in the number of circulating ILCs, but relatively ILC3 were most affected (108). Circulating ILCs also displayed decreased HLA-DR expression (108). This suggests that ILCs contribute to immunosuppression in sepsis. However, some pro-inflammatory capabilities seem to remain unimpaired as the ability to secrete TNF-α in response to TLR activation was not significantly affected (108). A decrease of ILC3 cells in septic patients was also reported in a prospective study comparing patients with septic shock, ICU patients without infection (trauma, cardiac arrest, neurological dysfunction) and healthy volunteers (109). But in this study, the decrease of ILC3 cells was accompanied by an increase in circulating ILC1 cells and a decrease in ILC2 cells (109). This also led to a relative decrease of ICL3 cells compared with ILC2 cells. The difference may be explained by different immune states of the septic patients included in these studies: While the second study only looked at septic patients with septic shock, the first study did not make such a distinction. We highlighted the potential role of the proportional changes of ILCs and summarized their functions in Figure 2.

Consequences for the role of ILCs other than NK cells in PICS—Based on these studies, particularly the latter, it can be postulated that ILC2 cells contribute significantly to the detrimental immunosuppressive state of patients with septic shock (109). Interestingly, TGF-β was found to promote the development of ILC2 cells and maintain their mature phenotype in the periphery in mice (110). As TGF-β can be upregulated in human trauma and sepsis (38, 111), this may promote ILC2-mediated immunosuppression. However, this needs to be confirmed in future studies.

Taken together, these studies suggest that ILCs play an important role in modulating the inflammatory response in a tissue- and immune-status-specific manner. Thus, to determine their role in acute sepsis, as well as their prolonged effects in PICS, it will be crucial to study ILCs’ tissue-specific responses.

### Natural killer T (NKT) cells

#### Types of NKT cells—

NKT cells are a heterogenous group of cells that have similarities to both T cells and ILCs: While they do express an antigen-receptor, they can be activated by antigen-independent mechanism, making them more “innate-like” than conventional T cells. Based on their antigen-receptor, NKT cells are classified as either invariant (iNKT/type I) or diverse (dNKT/type II). iNKT cells express the same invariant TCR α chain and share the same antigen specificity. dNKT cells, as the name indicates, can have a broader range of TCRs (112).

Effector functions of dNKT cells remain poorly characterized, whereas iNKT cells are known to contribute to the clearance of different bacteria, viruses, and fungi. iNKT cells usually reside in non-lymphoid tissues, where they can be recruited to sites of infection (113). They can be activated through both antigen-dependent and antigen-independent mechanisms. Antigen-mediated activation is driven by recognition of microbial lipids bound to CD1d receptors on APCs. Antigen-independent activation is driven by inflammatory cytokines (113). Upon activation, iNKT cells can kill cells directly either through engaging death ligands such as FasL and TRAIL, or releasing granzyme B and perforins (113). Moreover, a plethora of cytokines can be released by iNKT cells upon activation, including IFN-γ, IL-2, TNF-α, IL-4, IL-5, IL-10, IL-13, IL-17, IL-3, and granulocyte–monocyte colony-stimulating factor (GM-CSF). Depending on which cytokines are released and which receptors are expressed, iNKT cells can be sub-grouped similar to conventional T-cell lineages: NKT1 (IFN-γ), NKT2 (IL-4), NKT10 (IL-10), NKT17 (IL-17), and NKT_FH_ (follicular helper NKT cells, express follicle homing receptor CXCR5) (112). Additionally, another subset of iNKT cells has been described that is induced by TGF-β, expresses FoxP3, and has similar functions to Tregs (114). Lack of IL-12 production by myeloid cells drives NKT cells from IFN-γ to IL-4-producing subtypes (93). IL-4 and IL-10-producing subtypes can also be promoted through IL-10 or TGF-β (93) and experimentally through administration of altered lipid antigens (e.g., OCH) and immune checkpoint modulators (e.g., Tim-3 ligands) (112).

Two major functions in combatting extracellular bacterial infections can be exerted by iNKT cells: First, iNKT cells (NKT1) induce inflammation through release of IFN-γ to promote bacterial clearance. Second, iNKT cells have a regulatory role and promote resolution of inflammation through release of IL-4 (NKT2) or IL-10 (NKT10).

#### NKT cells in trauma and sepsis—

The release of IL-4 (NTK2) and IL-10 (NKT10) by iNKT cells promotes the shift to a Th2 cell-associated immune phenotype. This phenotype is thought to contribute to immunosuppression after sepsis (115, 116). IL-4 production was almost exclusively attributed to NKT cells in a burn model (93). Regulatory iNKT cell subsets could thus contribute substantially to immunosuppression during PICS; however, their exact roles remain to be elucidated.

The numbers of iNKT cells in peripheral blood are low, as they comprise only about 0.01% to 0.5% of all circulating T cells in humans. The percentages in blood are similar in mice (0.2%–0.5%), but mice have higher percentages of iNKT cells in the liver (< 0.4% in humans, ~30% in mice) (112).

#### Preclinical studies—

Murine sepsis studies have yielded contradictory results: Hepatic murine iNKT cells are activated and recruited to the peritoneum upon CLP and produce a wide array of pro-inflammatory cytokines, as well as IL-10. While two studies reported decreased mortality when iNKT cells were depleted, a study with CD1KO mice showed no effect of NKT cells on mortality (112, 117, 118). As the latter was the most severe CLP model, itis possible that NKT effects vary based on sepsis severity (112). Rather than depleting NKT cells, some studies have tried to skew the NKT cells response to achieve favorable outcomes in sepsis: administration of altered lipid antigens (e.g., OCH), cytokines (e.g., IL-30), and immune checkpoint modulators (e.g., Tim-3 ligands) shift iNKT cells toward the more regulatory subtypes and have demonstrated improved outcomes in experimental sepsis (119-121). In other mouse studies, activation of iNKT cells was reported to worsen the acute respiratory distress syndrome (ARDS) (122, 123), but to contribute to bacterial clearance in *Pseudomonas aeruginosa* and *Streptococcus pneumonia* (124, 125).

In terms of dNKT cells, a certain subtype was found to be protective after cardiac arrest and resuscitation (112), as well as following renal and hepatic ischemia-reperfusion injury (126, 127). Additionally, activation of dNKT cells by anti-inflammatory sulfatide was shown to lower mortality and production of inflammatory cytokines in a murine *Staphylococcus aureus* sepsis model (128).

In trauma, murine studies aimed to model the findings that in humans changes of NKT numbers in the acute trauma phase correlated with the development of multiple organ dysfunction syndrome (MODS) (44). The murine models showed that NKT cells are activated in the hyper-acute phase after trauma and hypothesize that this early mobilization influences long-term outcomes (129). Animal studies proving the causal connection between the acute activation and the development of MODS have yet to be conducted.

#### Clinical studies—

In septic patients, the absolute number of iNKT cells as well as the percentage of iNKT cells among T cells seems to increase slightly (112, 119, 130). However, this increase is not found in all populations of septic patients (112, 131). It remains to be elucidated whether this increase is accompanied by a phenotypical change toward an immunosuppressive iNKT cell type.

A recent study investigating post-sepsis immunosuppression found that increased IFN-γ serum levels in septic patients in the early stage of sepsis correlated with increased susceptibility to secondary Candida infection (30). This finding was attributed to iNKT-driven production of IFN-γ that impaired macrophage phagocytosis in a translational murine sepsis study (30). A second human sepsis study detected heightened activation of NKT cells with increased production of IL-4 and granzyme B (130), both of which can contribute to immunosuppression. On the other hand, the investigators also found increased IFN-γ levels (130). The study has some significant limitations as dynamics were not studied, the markers used to identify NKT cells are not specific for iNKT cells and potential phenotype changes were not assessed (130).

**Fig. 3.**
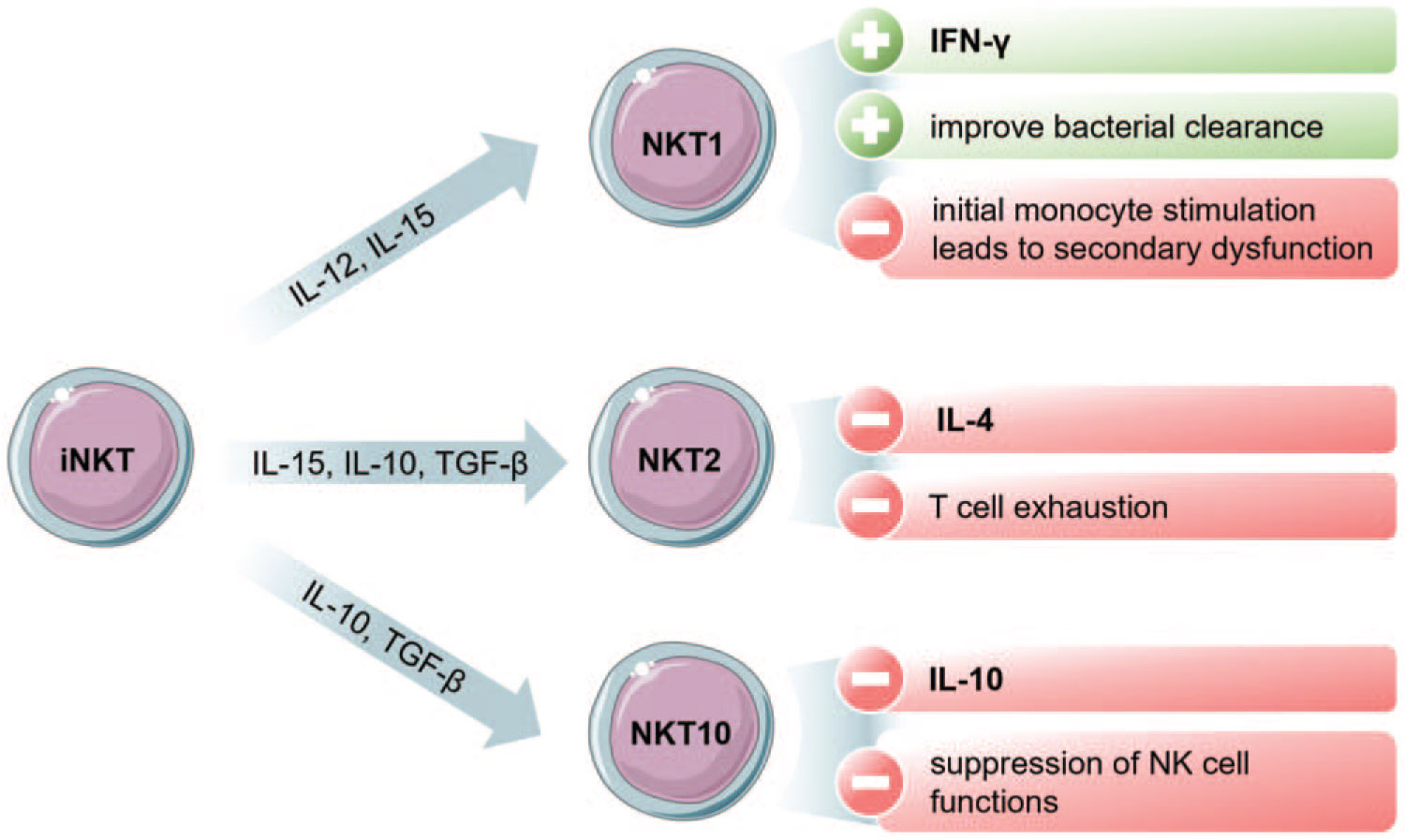
Different invariant natural killer T cells (iNKT) subsets fulfill different functions during inflammation. During post-septic or -traumatic inflammation iNKT can transform into different subsets, such as NKT1, NKT2, and NKT10 driven by IL-12, IL-15, TGF-β, and IL-10. The function of these subsets differs during inflammation: NKT1 mainly produces IFN-γ and promotes inflammation and bacterial clearance. The release of IL-4 (NTK2) and IL-10 (NKT10) by iNKT cells promotes the shift to a Th2 cell-associated immune phenotype, which is thought to contribute to immunosuppression.

Further studies are needed to clarify which iNKT cell-dependent mechanisms mainly contribute to immunosuppression in humans after sepsis. The suggested differentiation of NKT cells into immunosuppressive and pro-inflammatory subsets is visualized in Figure 3.

In trauma patients, only few studies examined the effect of NKT cells on systemic immune dysfunction. A study from Hazeldine et al. (44) found that the development of multiple organ dysfunction syndrome (MODS) is associated with elevated NKT numbers in the hyper-acute phase after trauma. Following up Jo et al. systematically examined the functionality of NKT cells in human traumatic injury. They displayed that in the acute phase of trauma NKT cells were relatively and absolutely decreased in numbers and impaired in the ability to proliferate and to produce IFN-γ when stimulated (132).

Both studies suggest that the initial impairment of NKT function contributes to the development of MODS (44, 132), which can be caused by chronic immune disturbances such as PICS (2). The studies did not differentiate between the different NKT subsets, which should be addressed in future studies.

Consequences for the role of NKT cells in PICS—Based on the preclinical and clinical studies and the known pro-inflammatory and regulatory roles of iNKT cells, it is possible that their effects are beneficial in the short term, when bacterial clearance is paramount, but detrimental in the long term when continued immunosuppression leaves the host susceptible to secondary infections. Thus, future studies should aim to examine the contribution of the different NKT subtypes and investigate whether the impairment persists chronically or if they contribute to the chronic dysfunction of other cells that might lead to the development of PICS. Moreover, future studies should include an assessment of bacterial loads and the immune status of the patient when determining the effects of the various iNKT subtypes and their effects on patient outcomes in sepsis and trauma.

### DN T cells

#### Types of DN T cells—

Double-negative T cells (DN T cells) express TCR-αβ, but neither CD4 nor CD8. They make up about 1% to 5% of the total T cell population in mice and humans (133-135). DNT cell activation can be antigen-driven (134). However, similar to NKT cells, co-receptor-independent activation mechanisms have also been suggested (136). The lack of appropriate distinguishing markers for DN T and NKT cells complicates investigation into their specific effects. Unsurprisingly, the described functions of DN T cells so far are thus quite similar to those of NKT cells: DN T cells produce similar cytokines to NK cells upon activation (IFN-γ, IL-4, IL-5, TGF-β, and IL-10 (135, 137, 138)), they can suppress CD4 and CD8 T cells, NK cells, dendritic cells, macrophages, B cells, and plasma cells and can also have cytotoxic effects through perforin/granzyme B and Fas/FasL-dependent pathways (135).

**Fig. 4.**
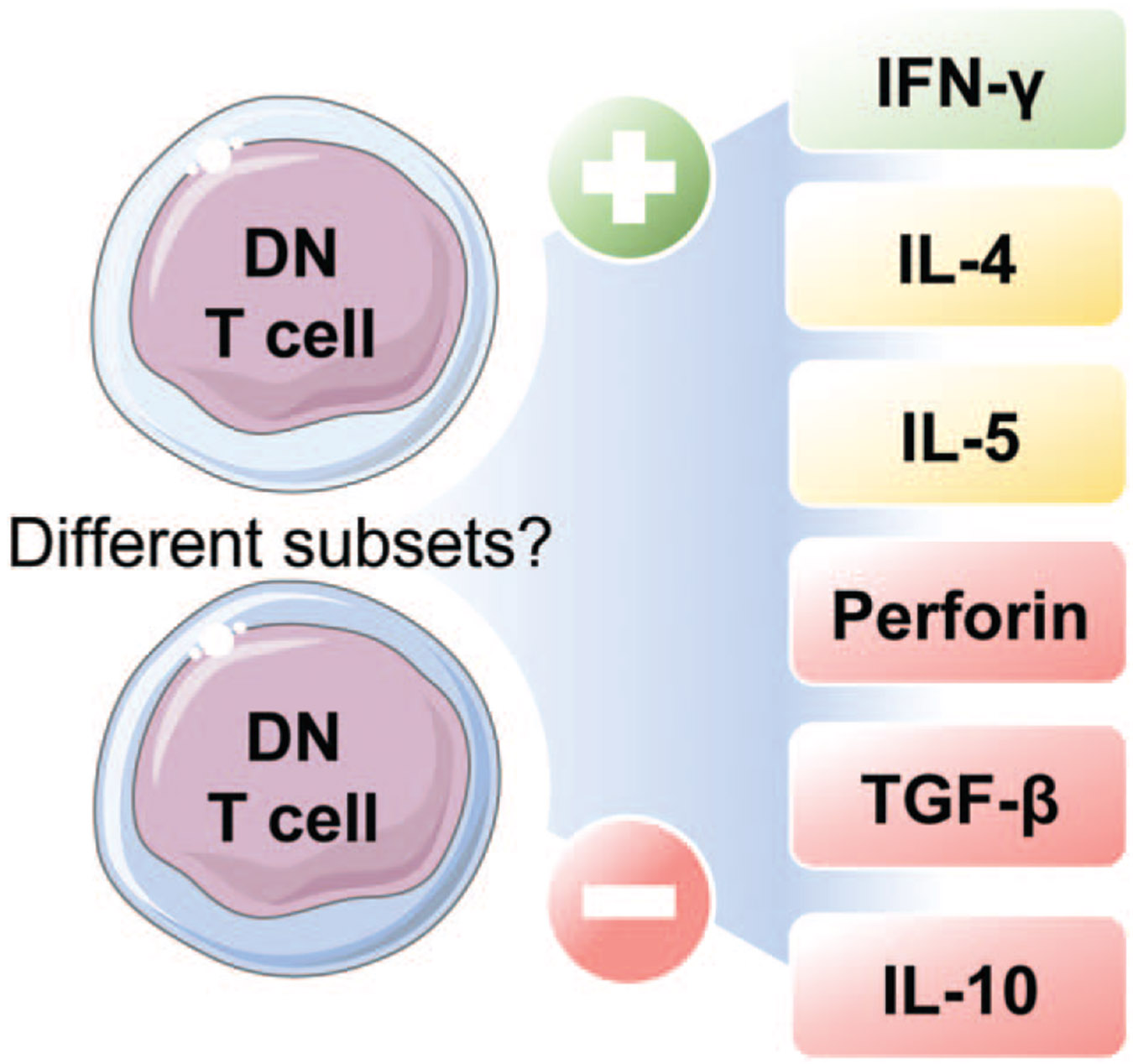
TCR-αβ^+^ double-negative T cells (DN T cells) are suggested to dichotomously affect inflammation. Double-negative T cells are known to produce IFN-γ, which is thought to be pro-inflammatory. But they also produce IL-4 and IL-5, which are usually attributed to a Th2 like immune response that characterizes impaired function after sepsis. Moreover, they produce perforin, TGF-β, and IL-10 which exert potent anti-inflammatory functions. Several studies suggest that DN T cells can be divided into suppressive and immune-stimulatory subtypes as they were shown to produce either IFN-γ or IL-10, but not both simultaneously. However, distinctive markers are currently missing to prove this suggested dichotomy.

Several studies suggest that DN T cells can be divided into suppressive and immune-stimulatory subtypes: In burn-injured mice, DN T cells were shown to contribute to post-burn immunosuppression through suppression of lymphocyte proliferation and production of IL-4 and IL-10, but they also produced IFN-γ and IL-2 (139). In septic mice, it was shown that DN T cells were either IFN-γ or IL-10 positive, but no dual-expressing DN T cells were found (140). This indicates that different DN T cell subsets may be distinguished based on their cytokine production. This suggested dichotomy is visualized in Figure 4. In line with this, a potentially distinct, third subset expressing IL-17A was described in a pulmonary infection model with *Francisella tularensis* live vaccine strain (141). Similar to the distinction from NKT cells, however, the lack of specific markers for these subsets complicates research into subtype-specific functions of different DN T cells.

#### DN T cells in trauma and sepsis—

Preclinical and clinical studies—Very little is known about how DN T cells can influence immunity after trauma and during sepsis and PICS. Infectious studies have shown that DN T cells increase at the site of infection (142) and potentially contribute to neutrophil recruitment (143). In a stroke model, DN T cells increased the numbers of pro-inflammatory microglia (144). It is unclear if DN T cells show similar dynamics and pro-inflammatory functions in trauma and acute sepsis. In a murine sepsis model, splenic DN T cell numbers were increased 1 week after CLP (140), which roughly corresponds to the time that mice develop PICS (20). The proportion of DN T cells even remained elevated at 1 month (140).

Consequences for the role of DN T cells in PICS—This indicates that DN T cells may contribute to the prolonged immune dysfunction in PICS in mice, but further studies in mice and humans are necessary to elucidate their specific role.

### B cells

#### Types of B cells—

Naive B cells are generally divided into B-1 B cells, follicular B cells, and marginal zone (MZ) B cells. The different subsets vary in terms of location, migration ability, and activation by T cell-dependent or -independent mechanisms. Studies on subset-specific functions have mostly been conducted in mice (145).

**Fig. 5.**
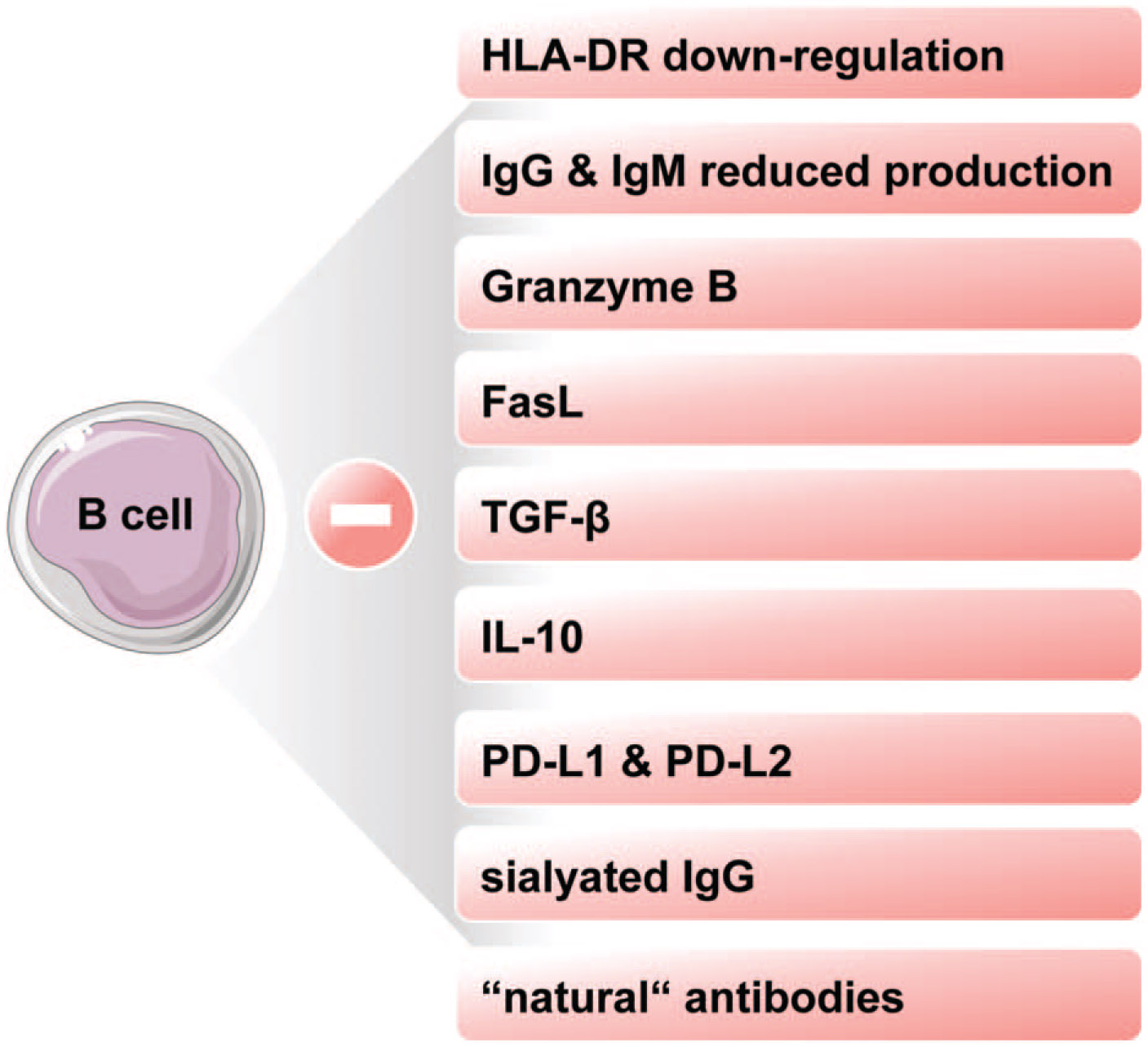
Immunosuppressive functions of B cells. B cells can exert a broad variety of immunosuppressive functions post-trauma and post-sepsis. Amongst these is the downregulation of HLA-DR, reduced IgG and IgM production, release of granzyme B, TGF-β, and IL-10 and the expression of FasL, PD-L1, and PD-L2. They also produce immunosuppressive antibodies: sialylated IgG antibodies have a reduced affinity for classic Fc-gamma receptors and mediate immunosuppression via a complex interaction with myeloid cells, impairing effector macrophages. Natural antibodies also exert immunosuppressive functions. They are low affinity antibodies that are believed to contribute to the elimination of apoptotic cells.

Immunosuppressive functions of B cells have only recently been studied. Mizoguchi et al. coined the term “regulatory B cells,” when they identified a discrete IL-10-producing population of B cells (146). However, there is no uniform categorization of these cells and their potential subtypes to date, but a number of Breg subsets with overlapping phenotypes and functions have been identified in various (mouse) models so far (147). Reported suppressive mechanisms include production of IL-10 and TGF-β (148, 149), release of cytotoxic granzyme B (149), expression of FasL (149), and induction of T-cell exhaustion through expression of PD-L1 and PD-L2 (148, 149). A mechanism unique to B cells is the production of immunosuppressive antibodies: sialylated IgG antibodies have a reduced affinity for classic Fc-gamma receptors and mediate immunosuppression via a complex interaction with myeloid cells, leading to the suppression of effector macrophages (reviewed in (149)). Additionally, “natural” antibodies have been attributed with strong immunosuppressive functions both *in vivo* and *in vitro*. Natural antibodies are low affinity antibodies produced by B-1 cells that are thought to help with elimination of apoptotic cells (149). The immunosuppressive functions are visualized in Figure 5. Finally, B-cells have been described to dampen inflammation by inducing CD4^+^ FoxP3^+^ regulatory T cells and Tr1 T cells (148).

#### B cells in trauma and sepsis—

Preclinical studies—In murine sepsis models, induction of B-cell lymphopenia was reported as well and began as early as 6 h after sepsis induction (150). Similar to observations from septic patients, lymphopenia in mice affected different subsets heterogeneously and there was actually an increase in the proportion of IL-10-producing regulatory B cells (150). These cells also displayed increased PD-L1 and decreased MHCII mRNA expression (150), indicating potential T-cell suppressive effects. In contrast, decreased function of regulatory B cells was suggested by decreased CD1d expression in a murine endotoxemia model and IL-10 expression by Bregs was decreased (151). The adoptive transfer of regulatory B cells from healthy wild-type mice was protective in this model and downregulated IFN-γ secretion in CD4 T cells (151). A transfer of cells from IL-10-deficient donors, however, did not confer protection (151). Of note, the role of regulatory B cells in endotoxemia appears to depend on the severity of the model, however, as an increase in regulatory B cells was reported in a mild endotoxemia model (151). This may also explain the contrasting findings in the CLP model.

In addition to the IL-10-producing subset, a new subset of B cells, which was coined innate response activator B cells, has been described in a murine sepsis model. These B cells acted in an innate, non-APC-induced manner and modulated the immune response through GM-CSF production (152).

#### Clinical studies—

An observational prospective clinical study including 52 patients revealed that, similar to T cells, B cells undergo apoptosis during septic shock (153). B-cell apoptosis during sepsis might lead to the production of anti-inflammatory cytokines by phagocytic cells (41, 153). B-cell lymphopenia was maintained throughout 28 days of follow-up (153). Of note, lymphopenia affected the B-cell subsets heterogeneously and septic shock survivors and non-survivors showed different B-cell patterns: A low percentage of CD23^+^ and a high percentage of CD80^+^ CD95^+^ B cells at ICU admission were associated with increased mortality (153). It has been reported that CD23 is expressed on activated B cells (153, 154), whereas CD80 is supposed to be a T-cell costimulation marker and CD95 indicates increased susceptibility to apoptosis (153). This indicates that CD23^+^ B cells represent functional and active cells beneficially contributing to the immune response, whereas CD80^+^ CD95^+^ B cells predict B-cell lymphopenia. Further indications that impaired B-cell function is detrimental in sepsis are the observation that levels of HLA-DR, a B-cell activation marker, are lower on B cells of trauma patients with severe sepsis compared to non-septic patients (155). Moreover, serum IgM levels in elderly septic patients (≥65 years) negatively correlate with acute physiology and chronic health evaluation II (APA-CHEII) score (156). The latter study also reported a reduced capacity for IgM production upon *ex vivo* stimulation in B cells of septic patients in general, as well as an increased proportion of exhausted B cells (CD21^−/low^) in septic patients compared to healthy controls (156). Septic patients were also reported to have significantly decreased serum gamma-immunoglobulin levels, which could contribute to increased susceptibility to secondary infections (157). Besides reduced production and/or secretion of gamma-immunoglobulins, vascular leakage due to endothelial damage, imbalanced distribution in inflamed tissues, disproportionate utilization by the complement system, and excessive catabolism have also been suggested as potential causes for hypo-gammaglobulinemia (158). Although the therapeutic benefit of polyclonal intravenous immunoglobulin (IVIG) therapy is disputed in sepsis, IVIG seems to have anti-inflammatory and anti-apoptotic effects on immune cells in addition to aiding in pathogen and toxin clearance (159).

#### Consequences for the role of B cells in PICS—

It is currently unclear what drives B-cell dysfunction in sepsis. It could be shown that TGF-β has immunomodulatory effects on B cells. It was reported to decrease proliferation of B cells and to increase apoptosis of immature or resting B cells in mice and humans (160-163). Furthermore, TGF-β can inhibit immunoglobulin synthesis and class switching in IgG isotypes and promotes the production of IgA in mice and humans (164-166).

Taken together, these studies suggest that B-cell dysfunction contributes to the development of a detrimental immunosuppressive state, but further research needs to be conducted to assess the clinical relevance of B-cell dysfunction in trauma, sepsis, and PICS. It remains to be elucidated whether A) the impairment of otherwise protective pro-inflammatory functions alone is responsible for worsened outcomes or B) immunosuppressive functions are triggered in sepsis that may play a detrimental role. Further, the role of B cells needs to be evaluated in PICS patients as well as using preclinical models of sustained immunosuppression to ascertain the underlying molecular mechanisms.

## DIAGNOSTICS AND POSSIBLE THERAPEUTIC INTERVENTIONS

The pathology of immune-perturbation in trauma, sepsis, and PICS is marked by the dichotomy of a robust pro-inflammatory upregulation of immunity as well as immunosuppression (167). Prior to administering therapy, it will be of utmost importance to assess the individual patient’s immune status to minimize the risk of adverse events.

It is important to highlight that over 30,000 patients over the past 30 years have been enrolled in clinical trials testing immune modulating treatments in sepsis (168), with none evidencing efficacy. These potential reasons for these failures can be illustrated by two prominent examples. A monoclonal antibody against TNF-α and anakinra (recombinant human IL-1-receptor antagonist) were both tested in clinical trials in septic patients, with both trials being terminated early due to ineffectiveness to reduce mortality (169, 170). However, in both cases later analyses showed that certain subgroups of patients benefited from the treatment. The antibody against TNF-α did reverse septic shock and deferred organ failure (171) and anakinra improved survival in patients with macrophage activation syndrome (172). We conclude that given the dynamic nature of sepsis and trauma, this necessitates an accurate assessment of the patients’ immune state and the degree of dysfunction, which can be made available in a timely manner and repeated easily to monitor disease progression and therapy response. This patient stratification is crucial and may allow future therapeutic approaches with immune modulators to be proven effective in reducing mortality and preventing PICS. Staging and personalized interventions for septic patients have recently been extensively reviewed (173, 174). Biomarkers that could be used to determine a patient’s immune status include cytokine levels, cellular cytokine production, and changes in leukocyte surface markers.

A plethora of studies examined cytokines for their predictive value in sepsis and trauma patients (12, 38, 111, 175). Studies that focused on multiple cytokines often demonstrated better predictive value (49, 50). In light of this, we postulate that the simultaneous assessment of several cytokines will be needed to properly evaluate a patient’s immune phenotype. Prominent pro-inflammatory cytokines that could be used to asses inflammation are TNF-α, IL-6, and IL-1β (12, 50, 175), whereas IL-10 and TGF-β are potent anti-inflammatory cytokines associated with disease severity and poor outcomes (49, 111, 176, 177).

Cytokine levels alone, however, do not sufficiently reflect the functional capacity of a patient’s cells (178). Flow cytometry can be used to gain preliminary insights. For example, T-cell activation is indicated by CD25, CD44, CD69, and CD71 positivity (179, 180), while activated monocytes upregulate HLA-DR and activated neutrophils CD64 (181-183). T-cell markers indicating a suppressed state include CD62L, PD-1, and cytotoxic T lymphocyte associated protein 4 (CTLA-4) (184, 185) and in the case of monocytes and neutrophils, the downregulation of CD88 (186) suggests poor responsiveness.

While flow cytometry can give us some insight, it is still a static measurement and does not adequately assess the function of a patient’s immune cells. Hence, to get a more complete picture, the use of functional assays has proven useful (51). A particularly promising technology is an enzyme-linked immune absorbent spot assay (ELISPOT). This method can report cellular responsiveness to physiologically relevant stimuli, is easy to perform and standardize, and the technology has already been cleared by the Food and Drug Administration (FDA). As ELISPOT can quantitate the total amount of a cell’s cytokine production over an extended period, it is more sensitive at revealing group differences in cell function compared with other methods using shorter incubation times (173).

If a patient is found to be in an immunosuppressed state by the above methods, his or her immune response could be augmented therapeutically. For example, cytokines could be used to boost adaptive and innate immune cells.

Currently, there are four cytokines that are being evaluated in clinical studies: IFN-γ, GM-CSF, IL-7, and IL-15. Blunted monocyte function in septic patients was successfully restored by IFN-γ, as determined by HLA-DR expression and *ex vivo* TNF-α secretion in response to stimulation (94, 187). The results of a phase III clinical study with IFN-γ are pending (NCT01649921). It was reported that GM-CSF increases HLA-DR expression on monocytes, restores TNF-α production in whole blood, and reduces the incidence of nosocomial infections in septic patients (184). Potent anti-inflammatory functions of GM-CSF are also described, including the expansion of myeloid-derived suppressor cells (188). Accurate immune stratification and close patient monitoring will therefore likely be of key importance for the success of GM-CSF therapy. A phase III clinical study is currently underway (NCT0261528). The survival and proliferation of T cells is promoted by IL-7, by increasing TCR diversity and augmenting T-cell recruitment to the site of infection in murine models, thereby promoting pathogen clearance and survival (184). Currently, a phase II study for the treatment of severe sepsis is underway (NCT02640807). IL-15 or an IL-15 super-agonist can be used to stimulate IFN-γ production by memory CD8^+^ T cells, NK cells, and NKT cells and thus improve their function and homeostasis. Unfortunately, severe toxic reactions, which were not seen with IL-7, occurred with IL-15 treatment. Thus, its broad clinical applicability in sepsis is questionable (189).

Other immunomodulatory therapies, originally developed for cancer, may also be useful for sepsis. PD-1, expressed on T cells, and its ligand PD-L1, expressed on monocytes and macrophages, constitute an immune checkpoint driving T-cell exhaustion and macrophage dysfunction (184, 185). The expression of PD-1 is elevated in septic patients and associated with increased mortality (190). In a murine model of polymicrobial sepsis, the use of anti-PD-1 restored T-cell apoptosis and improved survival (184, 191, 192). These promising results suggest that anti-PD-1 may have similar beneficial effects in septic patients. Another checkpoint receptor that was successfully targeted in murine models is CTLA-4. CTLA-4 is expressed on activated T cells and inhibits T-cell function upon binding to CD80 and CD86 on APCs (192, 193). Septic patients have increased CTLA-4 expression on CD4^+^ T cells (179, 184). In murine models, CTLA-4 inhibition diminished T-cell apoptosis (99) and was a suitable adjuvant to blocking PD-1 and PD-L1 (193).

A dichotomy of an immunosuppressive subset versus a pro-inflammatory subset was suggested for several of the lymphoid cells reviewed here (e.g., NKT10 vs NKT1 (115), ILC2 vs ILC3 (108, 109), IFN-γ^+^ vs IL-10^+^ DN T cells (140), CD80^+^ CD95^+^ B cells vs CD23^+^ B cells (153)). Ideally therapeutic interventions should aim to selectively skew populations toward a beneficial subtype and/or selectively deplete detrimental ones. However, it is important to consider the dynamic behavior as immunosuppressive subtypes might not drive the host immune response toward detrimental outcome at all times during the disease progression. Currently only IL-15 treatment augmenting NKT functioning in cancer patients (189) and the use of IL-7 to improve survival and proliferation of T cells (184) aim in this direction. Future studies examining subtype modulation in other lymphoid cells may improve and broaden the armamentarium of therapeutic approaches.

## CONCLUSION AND FUTURE DIRECTIONS

Sepsis is a heavy burden for every healthcare system and a challenge for every intensive care clinician, as it is responsible for one out of three in-hospital deaths (194). But even survivors are often unable to fully recover from sepsis and remain in a chronic state of immunosuppression called PICS. Not only septic patients can progress to PICS—it has been shown to develop after trauma as well (2, 31). As highlighted in this review, a number of different lymphoid cell populations have significant immunosuppressive functions that can contribute to this syndrome. However, despite a myriad of studies, there is no approved drug for the treatment of immune dysfunction in trauma and sepsis. With regard to the immunosuppressive functions reviewed in the previous chapters, we postulate that the following four measures will improve patient immunephenotyping and therapy development:

1. Continuous dynamic immune monitoring should be implemented as a standard in the ICU. It is not yet common clinical practice to assess a patient’s immunological state. However, based on his/her immune status at the time of intervention, the patient may need either an augmenting or a suppressive treatment—giving a booster to a patient who is already in a hyperinflammatory state or suppressing an already impaired immune system further will likely be detrimental. Thus, a “one size fits all” approach is unlikely to be successful. Academic ICUs should aim to evaluate cytokine levels and assess cellular activation markers and functional assays as their clinical diagnostic standards. The long-term goal should be obtaining a “live profile” of the immune response, comparable to the constant physiologic monitoring of ICU patients.
2. Several biomarkers and functional assays should be employed to determine the immunologic state, not just a single measurement. Studies show that single markers fail to predict outcomes in sepsis due to low sensitivity and specificity, but the use of ratios or combinations of several biomarkers improve sensitivity and specificity (49, 195, 196). The relatively new use of functional assays in combination with biomarkers may enhance this further and also improve the accuracy of predicting therapeutic responses (51). Thus, an immune status panel, rather than a single marker, can not only guide treatment decisions, but also improve analyses of clinical trials.
3. Subgroups in the septic and trauma patient populations must be defined based on their immune status. This patient stratification is crucial for clinical research, as many studies not only use different definitions for sepsis, which makes it difficult to compare results, but also broadly administer the tested treatment with no regard to a patient’s immune phenotype. Meta-analyses of some past clinical trials indicate that their failure was due to limiting the treatment to an appropriate patient population (172). A recently published retrospective analysis of 16,552 unique patients identifies four distinct clinical phenotypes that correlate with host-response patterns and clinical outcomes, and simulations suggest that these may help understand the heterogeneity of treatment effects (48).
4. Drugs developed for autoimmune diseases and cancer should be considered and tested for the treatment of immune dysfunction after sepsis and trauma. Autoimmune diseases and cancer share several immunological characteristics with sepsis immune dysfunction. It is therefore conceivable that immune-modulators developed for cancer or autoimmunity may also be beneficial for sepsis and trauma patients. Given that these drugs are already being tested in clinical trials and are potentially already FDA approved, they could be made available to sepsis and trauma patients in a timely manner, if a benefit in these patient populations can be demonstrated.

## Figures and Tables

**Table 1.** Characterization of the Treg family

Origin	Treg type	Markers	Cytokines produced	Cytokines inducing cell type	Function and tissue residency
tTregs	Th1 like Treg	Surface cell receptors	IFN-γ	Ag + IL-2	Colon, skin
		TCR, CD2, CD25 CD45RO, CD127^low^, Helios, CTLA-4	IL-21	Ag + IL-12/ IFN-γ	
			IL-22		
		Transcription factors	little IL-10		
		FoxP3, T-bet, Stat1			
		Chemokine receptor			
		CXCR3, IFNGR, IL-12Rβ2			
	Th2 like Treg	Surface cell receptors	IL-5	Ag + IL-2 Ag + IL-5	Thymus, spleen, liver, skin
		TCR, CD2, CD25, CD45RO, CD127^low^, Helios, CTLA-4	IL-4		
			IL-4		
		Transcription factors	IL-10		
		FoxP3, Gata3	IL-13		
		IRF4, Stat4	no IL-22		
		Chemokine receptor			
		CCR8			
		IL-5Rα			
	Th17 like Treg	Surface cell receptors	IL-17A	IL-6, IL23, IL-1β	Spleen, liver, blood, skin
		TCR, CD2, CD25			
		CD45RO, CD127^low^	IFN-γ		
		Helios, CTLA-4	IL-21		
		Transcription factors	IL-10		
		FoxP3, RORγt			
		Stat3			
		Chemokine receptor			
		CCR6			
	Th22 like Treg	Surface cell receptors	little IL-10	?	?
		TCR, CD3, CD25			
		CD45RO, CD127^low^	no IL-22		
		Helios, CTLA-4	no IL-4		
		Transcription factors			
		FoxP3			
		Chemokine receptor			
		CLA^hi^			
pTregs	Tr1	Surface cell receptors	IL-10	IL-10	Systemic suppressor
	T cell	LAG-3	TGF-β		
		CD49b			
		CD25^−^	little IFN-γ		
		CTLA-4	little IL-2		
		Transcription factors	little IL-5		
		FoxP3^−^			
	Th3	Surface cell receptors	TGF-β	TGF-β	Mucosal suppression
		CD25^−^		IL-10	
		CTLA-4		Induced by specialized APCs	
		Transcription factors			
		FoxP3^+/−^			
	iTreg	Surface cell receptors	IL-10	TGF-β and presence of Ag	Ag specific suppression
		CD25	TGF-β		
		Transcription factors			
		FoxP3			

Ag indicates antigen; APC, antigen-presenting cell; iTreg, induced regulatory T cell; pTregs, peripherally derived regulatory T cells; Th like Tregs, T helper cell like regulatory T cells; Tr1 T cells, type 1 regulatory T cells and tTregs: thymus-derived regulatory T cells.

The table was compiled from (70, 197-200).

**Table 2.** Functional differentiation of Tregs

	Markers mice	Markers humans	Function
“Central” cTregs	CD62L^hi^	FoxP3^+^	Circulating in blood circulation and secondary lymphoid organs
	CD44^low^	CD25^hi^	
	CCR7^hi^	CD45RA^+^	Limited immunosuppressive capacity
	CD103^−^	CD45RO^−^	
	FoxP3^+^	CD62L^hi^, CD44^hi^	
	CD25^hi^	CD127^low^	
	ICOS^low^		
	CTLA-4^indifferent^	CD69^low^	
	CD127^low^	CTLA-4^low^	
	Ki67^low^	ICOS^low^	
	BCL-2^hi^	HLA-DR^low^	
		BCL-2^hi^	
		CCR7^hi^	
		Ki67^low^	
“Effector” eTregs	CD62L^low^	FoxP3^+^	Considered “activated” cTreg
	CD44^hi^	CD25^hi^	In comparison the cTregs more abundant in nonlymphoid tissue
	CCR7^low^	CD45RA^−^	
	CD103^+^	CD45RO^+^	Suppressive function considered more potent than in cTregs.
	ICOS^hi^	CD62L^low^, CD44^hi^	
	FoxP3^+^	CD127^low^	
	CD25^indifferent^	CD69^hi^	
	ICOS^hi^	CTLA-4^hi^	
	CTLA-4^hi^	ICOS^hi^	
	CD127^low^	HLA-DR^hi^	
	Ki67^hi^	BCL-2^low^	
	BCL-2^low^	CCR7^low^	
		Ki67^hi^	
“Memory” mTreg	CD62L^low^	FoxP3^+^	High immunosuppressive capacity
	CD44^hi^	CD25^hi^	Mostly abundant in peripheral tissue with a long life span
	CCR7^low^	CD45RA^−^	
	FoxP3^+^	CD45RO^+^	
	CD25^hi^	CD62L^low^, CD44^hi^	
	CTLA-4^hi^	CD127^low^	
	CD127^hi^	CCR7^low^	
		CD27^hi^	
		CTLA-4^hi^	
		ICOS^hi^	
		BCL-2^hi^	
		Ki67^low^	
Tissue resident Tregs	FoxP3^+^, T-bet^+^, GATA3^+^, CXCR3^+^, CCR4^+^	?	Control skin and lung inflammation
	FoxP3^+^, Stat3^+^, CCR6^+^	?	Control gut homeostasis
	◯ RORγt^−^, Helios^−^,Nrp1^−^		
	◯ RORγt^+^, Helios-,Nrp1^−^		tolerance toward dietary antigens
	◯ GATA3^+^, Helios^+^,ST2^+^		tolerance toward small intestinal microbes
			tissue repair
	FoxP3^+^, BCL-6^+^, CXCR5^+^	?	Control germinal centers
	FoxP3^+^, PPARγ^+^, IRF4^+^, BATF^+^, CCR2^+^, specific TCR repertoire	?	Control adipose metabolism
	FoxP3^+^, Stab1^low^, ST2^high^, CCR2^+^, CTLA-4^+/ high^, TIM-3 ^high^, repeated TCR repertoire	?	Potentiate muscle repair

The table was compiled from (68, 84, 201-205).

**Table 3.** Nomenclature and function of innate lymphoid cells (ILCs)

ILC type	Markers	Cytokines produced	Stimuli inducing cell type	Function and major tissue residency
NK, ILC1	Transcription factors	IFN-γ	Tumors, intracellular microbes	Type 1 Immunity
	T-bet (NK: EOMES, ILC1: NFIL3, RUNX3)	TNF-α		NK: Blood, liver
		Granzymes		ILC1: Lung, intestines, mesenteric lymph nodes, tissue resident, Spleen
		Perforin		
ILC2	Transcription factors	IL-4	Large extracellular parasites and allergens	Type 2 Immunity
	Gata3, RORα, Bcl11b, GFI1	IL-5		Lung, intestines, mesenteric lymph nodes, spleen
		IL-13		
		IL-9		
		AREG		
ILC3	Transcription factors	IL-22	Extracellular microbes	Type 3 Immunity
	RORγt, AHR, ID2	IL-17		Intestine, mesenteric lymph nodes, spleen
		GM-CSF		
		Lymphotoxin		
LTi	Transcription factors RORγt, TOX, ID2	TGF-β	Mesenchymal organizer cells	Formation of secondary lymphoid structures?

LTi indicates lymphoid tissue inducer cells; NK, natural killer cells.

The table was compiled from (85, 101, 206).
